# Association of angiotensin-converting enzyme inhibitor therapy and comorbidity in diabetes: results from the Vermont diabetes information system

**DOI:** 10.1186/1472-6823-8-17

**Published:** 2008-12-05

**Authors:** Maria E Ramos-Nino, Charles D MacLean, Benjamin Littenberg

**Affiliations:** 1 University of Vermont, Department of Pathology, Burlington, Vermont, USA; 2 University of Vermont, General Internal Medicine, Burlington, Vermont, USA; 3 University of Vermont, Department of Nursing, Burlington, Vermont, USA

## Abstract

**Background:**

Angiotensin converting enzyme inhibitors (ACE inhibitors) reduce peripheral vascular resistance via blockage of angiotensin converting enzyme (ACE). ACE inhibitors are commonly used to treat congestive heart failure and high blood pressure, but other effects have been reported. In this study, we explored the association between ACE inhibitor therapy and the prevalence of comorbid conditions in adults with diabetes

**Methods:**

We surveyed 1003 adults with diabetes randomly selected from community practices. Patients were interviewed at home and self-reported their personal and clinical characteristics including comorbidity. Current medications were obtained by direct observation of medication containers. We built logistic regression models with the history of comorbidities as the outcome variable and the current use of ACE inhibitors as the primary predictor variable. We adjusted for possible confounding by social (age, sex, alcohol drinking, cigarette smoking) and clinical factors (systolic blood pressure, body mass index (BMI), glycosolated hemoglobin (A1C), number of comorbid conditions, and number of prescription medications).

**Results:**

ACE users reported a history of any cancer (except the non-life-threatening skin cancers) less frequently than non-users (10% *vs*. 15%; odd ratio = 0.59; 95% confidence interval [0.39, 0.89]; *P *= 0.01); and a history of stomach ulcers or peptic ulcer disease less frequently than non-users (12% *vs*. 16%, odd ratio = 0.70, [0.49, 1.01], *P *= 0.06). After correcting for potential confounders, ACE inhibitors remained significantly inversely associated with a personal history of cancer (odds ratio = 0.59, [0.39, 0.89]; *P *= 0.01) and peptic ulcer disease (odd ratio = 0.68, [0.46, 1.00], *P *= 0.05).

**Conclusion:**

ACE inhibitor use is associated with a lower likelihood of a history of cancer and peptic ulcers in patients with diabetes. These findings are limited by the cross sectional study design, self-report of comorbid diagnoses, and lack of information on the timing and duration of ACE inhibitor use. Further research is needed to confirm these associations and understand their mechanisms.

## Background

Although introduced for the treatment of hypertension, angiotensin converting enzyme (ACE) inhibitors are also able to reduce morbidity and mortality in congestive heart failure and post myocardial infarction, and to prevent re-infarction (reviewed in [1]), but have been found to have other benefits. For example, ACE inhibitors have been observed to slow the progression of diabetic nephropathy [2,3]. Lever *et al*. [4] found that users of ACE inhibitors exhibited a reduced risk of cancer (RR = 0.63; 95% CI, 0.41–0.93). Koh *et al*. [5] found that individuals possessing low-activity ACE alleles were at a reduced risk of breast cancer, suggesting that inhibition of the angiotensin II effect by blockade of ACE could be potential targets for the prevention and treatment of breast cancer.

Diabetes increases the risk of vascular complications [6]. Evidence from large-scale clinical trials has suggested that attenuation of the renin-angiotensin-aldosterone system by ACE inhibitors may reduce cardiovascular morbidity and mortality in diabetic patients with established cardiovascular disease [7-9]. ACE inhibitors may also increase survival in diabetes patients without heart disease and some large studies have shown that ACE inhibitors were able to reduce the risk of developing diabetes itself [10]. Here, we report the association between ACE inhibitor therapies and cancer or stomach/peptic ulcer disease in diabetic patients.

## Methods

This study is part of a larger project, the Vermont Diabetes Information System (VDIS), a study of 8,855 adults with diabetes in primary care practices [11]. The subjects comprised all diabetic adults in 69 practices in Vermont and adjacent New Hampshire and New York. A field survey was completed at study baseline in a sub-sample of subjects. Patients' names were randomly sorted and patients contacted by telephone until approximately 15% of patients from each practice agreed to participate in the field survey. Four patients were dropped from the analysis due to incomplete information leaving a final sample of 1,003.

Subjects completed a questionnaire at home and were visited by a trained research assistant who reviewed the questionnaire responses, assisted the subject with any missing or unacceptable responses, reviewed the subject's medications, and measured their blood pressure, height and weight using a portable sphygmomanometer, stadiometer, and scale. Race, education, income, marital status, functional status, smoking, alcohol consumption, and comorbid conditions were obtained by questionnaire. Prior to the interview, patients were instructed to gather all current medications, including over the counter preparations, for review by the research assistant. The medication list was ascertained by direct observation of the medication container with recording of the drug name, dose, frequency, and route of administration. Duration of therapy was not recorded.

To determine comorbidity, we used a modification of the Self-Administered Comorbidity Questionnaire [12] in which we asked each patient to indicate whether they have had the following conditions: coronary artery disease (CAD), congestive heart failure (CHF), peripheral vascular disease (PVD), cerebrovascular accident or stroke (CVA), Alzheimer or any other dementia, asthma/chronic obstructive lung disease, rheumatic disease (rheumatoid arthritis, lupus or polymyalgia rheumatica), stomach ulcers or peptic ulcer disease (PUD), cirrhosis, paralysis, renal insufficiency, microvascular complications (eye, nerve, kidney damage related to diabetes), AIDS/HIV, and depression. All patients had diabetes which was not included in the comorbidity count. Patients were classified as having cancer if they reported any non-skin cancer including leukemia or lymphoma. Specific cancer sites and dates of diagnosis were not recorded.

The interviews occurred between July 2003 and March 2005. Most laboratory data were obtained from the patients' local clinical laboratories, which all use the same Diabetes Control and Complications Trial/Epidemiology of Diabetes Interventions and Complications high performance, liquid chromatography (HPLC) method for the determination of glycosylated hemoglobin (A1C). Less than 1% of A1C tests were done using the Bayer DCA 2000 immunoassay point of care instrument, which has been shown to compare favorably with the HPLC method [13].

The research protocol was carried out in compliance with the Helsinki Declaration and was approved by the Committee on Human Research of the University of Vermont. The interviewed subjects provided written informed consent. The full study protocol and variables, and the medication profiles of the subjects have been previously reported [11,14].

We performed a cross sectional analysis of the interviewed subjects at the time of their enrollment in the VDIS trial. We explored the association between rheumatic disease, asthma/COPD, CAD, cancer, CHF, cirrhosis, CVA, depression, paresis, PUD, PVD, and the use of ACE inhibitors therapy using logistic regression with each condition as the outcome variable and the use of ACE inhibitors as the primary predictor variable. We then adjusted for possible confounding by social and clinical factors including gender, age (years), systolic blood pressure (mmHg), glycosylated hemoglobin level (A1C in mg%), body mass index (BMI in kg/m^2^), current alcohol use (yes/no), current cigarette use (yes/no), number of comorbidities, and number of prescription medications. The selection of these potential confounding conditions was based on clinical and epidemiologic judgment and not on statistical determinants. We used Stata/SE 9.2 (StataCorp, College Station, TX) for all analyses.

## Results

The study population was representative of adults with diabetes in primary care practices in Northern New England. See Table 1.

**Table 1:** Baseline characteristics of 1,004 adults with diabetes.

*Characteristic*	*n (%) or mean (sd)*
Ace Inhibitors use	445 (44.3%)
Men	457 (45.5%)
Age, years	64.8 (12.0)
Systolic Blood Pressure, mmHg	140.3 (19.6)
Glycosolated hemoglobin A1C, %	7.12 (1.3)
Body mass index, kg/m^2^	33.81 (7.4)
Alcohol use	274 (27.4%)
Cigarette smoking	170 (17.0%)
Number of comorbid conditions	1.8 (1.7)
Number of prescription medications	6.7 (3.8)
Rheumatic disease	142 (14.2%)
Asthma/chronic obstructive lung disease	203 (20.2%)
Coronary artery disease	193 (19.2%)
Cancer	126 (12.6%)
Congestive heart failure	172 (17.2%)
Cirrhosis	18 (1.8%)
Stroke	118 (11.8%)
Depression	351 (35.0%)
Foot ulcers	111 (11.2%)
Foot/leg pain	291 (31.0%)
Microvascular disease	170 (17.0%)
Paresis	30 (3.0%)
Peptic ulcer disease	143 (14.3%)
Peripheral vascular disease	88 (8.8%)
Renal problems	50 (5.0%)
Sexual dysfunction	244 (26.6%)
Stomach emptying	56 (6.2%)

sd = standard deviation; n = number of subjects with the characteristic

Table 2 presents the univariate associations between each comorbid condition and the use of ACE inhibitors. Cancer and PUD were significantly associated with ACE inhibitors therapy. Table 3 presents the univariate association between ace inhibitor therapy and other patient characteristics. Ace inhibitor use is significantly associated with being male, having higher glycosylated hemoglobin level (A1C), having higher body mass index (BMI, kg/m^2^), having higher number of prescription medications, and being a non-drinker.

**Table 2:** Univariate associations between comorbidity and ACE inhibitor therapy.

*Comorbidity*	*ACE inhibitor non- users* *n (%)*	*ACE inhibitor users* *n (%)*	*Odds Ratio*	*P*
Rheumatic disease	80 (14.3)	62 (13.9)	0.97	0.86
Asthma/COPD	116 (20.8)	87 (19.6)	0.93	0.63
CAD	98 (17.6)	95 (21.4)	1.27	0.13
**Cancer**	**82 (14.7)**	**44 (9.9)**	**0.64**	**0.02**
CHF	87 (15.6)	85 (19.1)	1.28	0.14
Cirrhosis	9 (1.6)	9 (2.0)	1.26	0.63
CVA	70 (12.5)	48 (10.8)	0.84	0.39
Depression	198 (35.5)	153 (34.4)	0.95	0.72
Paresis	17 (3.1)	13 (2.9)	0.96	0.91
**PUD**	**90 (16.1)**	**53 (11.9)**	**0.70**	**0.06**
PVD	45 (8.1)	43 (9.7)	1.22	0.38

N = 1003

**Table 3:** Univariate associations between ACE inhibitor therapy and other patient characteristics.

*Characteristic*	*ACE inhibitor users* *% or mean (sd)*	*ACE inhibitor users* *% or mean (sd)*	*Odds Ratio*	*P*
Number of subjects	558	445		
Age, years	65.2 (12.1)	64.3 (11.9)	0.99	0.24
Men	41.5%	50.6%	1.44	0.004
Systolic Blood Pressure	139.7 (18.8)	141.0 (20.5)	1.00	0.28
Glycosolated	7.1 (1.3)	7.2 (1.3)	1.10	0.05
hemoglobin A1C, %				
Body mass index, kg/m2	33.2 (7.3)	34.6 (7.4)	1.03	0.002
Alcohol drinking	30.2%	23.8%	0.72	0.03
Cigarette smoking	18.5%	15.1%	0.78	0.15
Comorbidity conditions	1.81 (0.1)	1.83 (0.1)	1.01	0.82
Number of prescription meds	6.2 (3.9)	7.2 (3.5)	1.10	<0.0001

Univariate associations between potential confounding variables and cancer were evaluated (Table 4) and a logistic regression model constructed using cancer as the outcome and all potential confounders: gender, age (years), systolic blood pressure (mmHg), glycosylated hemoglobin level (A1C) (mg%), body mass index (BMI, kg/m^2^), alcohol drinking (yes/no), cigarette smoking (yes/no), number of comorbidities, and number of prescription medications. This model showed a significant association between cancer and the use of ACE inhibitors (OR = 0.59, 95%CI [0.39, 0.89], *P *= 0.01) (Table 5). Other variables significantly associated with cancer history included age and number of prescription medications (Table 5).

**Table 4:** Univariate associations between cancer and other patient characteristics.

*Characteristic*	*Cancer Patients* *% or mean (sd)*	*Non-Cancer Patients* *% or mean (sd)*	*Odds Ratio*	*P*
Number of subjects	126	877		
ACE inhibitor therapy	34.9%	45.7%	0.64	0.02
Age, years	69.1 (10.2)	64.2 (12.1)	1.04	<0.001
Male	42.1%	46.1%	0.85	0.40
Systolic Blood Pressure, mmHg	143.0 (20.9)	139.9(19.4)	1.01	0.10
Body mass index, kg/m^2^	32.6 (6.8)	34.0 (7.5)	0.97	0.06
Alcohol drinking	25.4%	27.6%	0.89	0.60
Cigarette smoking	11.1%	17.8%	0.58	0.06
Number of comorbid conditions	1.8 (2.0)	1.7 (1.6)	1.05	0.41
Number of prescription medications	7.3 (4.3)	6.6 (3.7)	1.05	0.05

**Table 5:** Multivariate associations between cancer and other patient characteristics

*Characteristic*	*Odds Ratio*	*P*
ACE inhibitor therapy	0.59	0.01
Age, years	1.02	0.02
Male	0.88	0.56
Systolic Blood Pressure, mmHg	1.01	0.28
Glycosolated hemoglobin A1C, %	0.95	0.52
Body mass index, kg/m^2^	0.98	0.15
Alcohol drinking	1.07	0.77
Cigarette smoking	0.68	0.22
Number of comorbid conditions	0.97	0.61
Number of prescription medications	1.09	0.01

N = 974

Similarly, Table 6 presents the univariate associations between PUD and potential confounding variables. The logistic regression model showed a significant negative association with ACE inhibitors use (OR = 0.68, 95%CI [0.46, 1.00],*P *= 0.05) (Table 7). Other variables significantly associated with PUD history included alcohol drinking and comorbid conditions.

**Table 6:** Univariate associations between PUD and other patient characteristics.

*Characteristic*	*PUD Patients* *% or mean (sd)*	*Non-PUD Patients* *% or mean (sd)*	*Odds Ratio*	*P*
Number of subjects	143	860		
ACE inhibitor therapy	37.1%	45.6%	0.70	0.06
Age, years	65.0 (11.8)	64.8 (12.0)	1.00	0.83
Male	43.4%	45.9%	0.90	0.57
Systolic Blood Pressure, mmHg	137.7 (19.5)	140.7(19.6)	1.00	0.09
Glycosolated hemoglobin A1C, %	7.1 (1.3)	7.1 (1.3)	1.00	0.71
Body mass index, kg/m^2^	34.6 (7.4)	33.7 (7.4)	1.01	0.16
Alcohol drinking	14.7%	29.5%	0.41	<0.001
Cigarette smoking	24.5%	15.7%	1.74	0.01
Number of comorbid conditions	2.5 (1.8)	1.5 (1.5)	1.4	<0.001
Number of prescription medications	8.0 (4.2)	6.4 (3.7)	1.11	<0.001

**Table 7:** Multivariate associations between PUD and other patient characteristics

*Characteristic*	*Odds Ratio*	*P*
ACE inhibitor therapy	0.68	0.05
Age, years	1.00	0.96
Male	1.15	0.49
Systolic Blood Pressure, mmHg	0.99	0.23
Glycosolated hemoglobin A1C, %	0.94	0.40
Body mass index, kg/m^2^	1.01	0.57
Alcohol drinking	0.47	0.005
Cigarette smoking	1.51	0.09
Number of comorbid conditions	1.32	<0.001
Number of prescription medications	1.01	0.68

N = 974

## Discussion

These data suggest a negative association of ACE inhibitor use with both cancer and PUD in a community based population of adults with diabetes.

Evidence from animal models suggests that angiotensin II stimulates neovascularization [17] and promote angiogenesis in neoplastic growth. Angiotensin II may also act as a mitotic factor by inducing or regulating gene expression in cell cycle progression [18]. The ACE inhibitor captopril has been shown to inhibit proliferation of a variety of cell types, including human breast cancer [16,19,20], and to reduce tumor growth in experimental models of cancer [21,22]. Some epidemiological evidence also suggests protective effects of ACE inhibitors on cancer [4,5]. Here we present additional evidence of a potential negative association between cancer and the use of ACE inhibitors in a diabetic population.

Very few reports have looked at the association between ACE inhibitors and peptic ulcers. One study found that captopril significantly reduced the development of gastric ulcers in pylorus-ligated rats [23]. Another found that captopril and naloxone significantly reduced ulcer formation induced by stress in rats [24]. Mou *et al*. [25] determined that endogenous angiotensin II plays a role in the development of stress ulcers in rats with obstructive jaundice and that ACE inhibitors prevented their development. A report by Iakubov and Usmanova [26] indicated that prophylactic use of ACE inhibitors reduced indomethacin-induced ulcers and erosions of the stomach in humans. They suggested that the reparative functions of the mucous of the gastrointestinal track were influenced by a reduction of angiotensin II formation and activation of the renin-kallicrein-kinin system. These animal studies support the findings that ACE inhibitors may protect against PUD. We are aware of no prior studies of the association between ACE inhibitors and PUD in humans, but Sugimoto *et al*. [27] suggested that ACE gene polymorphisms were associated with cancer risk and gastric ulcers in Japan.

The mechanisms of the observed associations between ACE inhibition and the two diagnoses are unknown. It is possible that ACE inhibitors protect against the development of cancer or ulcer disease, although confirmation of these hypotheses must await additional investigation. Another interpretation of our results is the "treatment-risk paradox" in which the history of cancer or ulcer disease inhibits the use of ACE inhibitors, perhaps due to concerns of polypharmacy or intolerance of the medication due to the underlying disease. However, the prescribing information for ACE inhibitors does not discourage their use in either cancer or PUD and univariate analysis shows no significant differences between the number of comorbidities in ACE inhibitor users and non-users. These facts argue against the "treatment-risk paradox" theory, although not conclusively.

This study has several strengths. First, the interviewed subjects were a randomly selected subset of a large population of patients receiving care in the Northeast, and are therefore likely to be representative of primary care patients generally. Second, all data on medications were obtained by direct observation in the patient's home and not from secondary sources. Third, multiple potential confounders were assessed and found to have no significant effect on the apparent associations between ACE inhibition and PUD and cancer.

This study has several limitations including lack of clinical confirmation of the comorbid diagnoses, and lack of information on the timing and duration of ACE inhibitor treatment relative to the onset of the comorbidities. As in any cross-sectional study, unmeasured confounders may be responsible for the apparent associations found.

## Conclusion

These data suggest a potential protective association of ACE inhibitors for cancer and peptic ulcer disease in patients with diabetes. Further research is needed to confirm these associations and understand their mechanisms.

## Competing interests

The authors declare that they have no competing interests.

## Authors' contributions

MERN, CDM and BL contributed equally to all aspects of this work.

## Pre-publication history

The pre-publication history for this paper can be accessed here:


